# Comparative Study of Preoperative Urine Culture and Intraoperative Stone Culture in Predicting Postoperative Morbidity

**DOI:** 10.7759/cureus.92522

**Published:** 2025-09-17

**Authors:** Vinay S Kundargi, Gulshan Kumar, Siddanagouda B Patil, Santosh Patil

**Affiliations:** 1 Urology, Shri B.M. Patil Medical College Hospital and Research Centre, Bijapur Lingayat District Educational Association (Deemed to be University), Vijayapura, IND

**Keywords:** postoperative morbidity, stone culture, urine culture, urolithiasis, urosepsis

## Abstract

Introduction: Postoperative urosepsis after endourological treatments is a life-threatening condition, especially when the individual has urolithiasis. Although preoperative urine culture is a routine tool for guiding antibiotic prophylaxis, it frequently fails to identify bacteria embedded in urinary stones, which may lead to undiagnosed postoperative morbidity. Hence, this study aimed to evaluate the association between preoperative urine culture and intraoperative stone culture and determine their predictive value for postoperative infectious complications in patients undergoing endourological procedures.

Material and methods: This prospective observational study was conducted among 125 adult patients undergoing surgical treatment of urolithiasis in a tertiary care facility between June 2023 and December 2024. Urine culture collected before surgery and stone for stone culture obtained during surgery were tested for microbial growth and antibiotic sensitivity patterns. C-reactive protein (CRP), neutrophil-to-lymphocyte ratio (NLR), total leukocyte count (TLC), and hospital stay were measured as postoperative parameters in addition to culture results.

Results: Culture was positive in the urine of 27 (21.6%) patients, while stone culture was positive in 61 (48.8%) cases. The most common organisms were *Escherichia coli* (*E. coli*), *Pseudomonas*, and *Klebsiella*. A non-significant correlation existed between urine and stone culture (p > 0.05). Patients with positive stone culture had markedly elevated postoperative inflammatory markers and higher morbidity rates, irrespective of urine culture results.

Conclusion: Preoperative urine culture alone is an unreliable predictor of postoperative infectious complications. Intraoperative stone culture is a superior tool for identifying occult pathogens and predicting morbidity. Incorporating routine stone culture into urolithiasis management can enable timely, targeted antibiotic therapy and reduce postoperative morbidity.

## Introduction

Urolithiasis is a global health concern, with increasing prevalence rates worldwide. It is estimated to affect about 12% of the world population at some point in their lifetime [1]. There are various endourologic options for calculus clearance, like extracorporeal shock wave lithotripsy (ESWL), ureterorenoscopic lithotripsy (URSL), percutaneous nephrolithotomy (PCNL), and retrograde intrarenal surgery (RIRS) [1,2]. Even with advancements in technology, there is an increasing incidence of urosepsis [3,4]. Urosepsis refers specifically to sepsis resulting from a urinary tract infection (UTI) and is a potentially fatal systemic response. These infections are commonly caused by bacteria introduced during procedures like catheterization, following ureteroscopy, or other urinary tract manipulation [1,2]. There are several risk factors for urosepsis, including prolonged catheterization, poor hygiene practices, and immunocompromised status. For a long time, infection has been known to be associated with urinary calculi [2].

After stone manipulation or fragmentation, it is not uncommon for patients with a sterile preoperative urine culture to develop postoperative sepsis, with reported incidence rates ranging from approximately 3% to 6%, possibly due to bacterial release into the bloodstream. For instance, in a study of endourological procedures, around 3% of patients developed sepsis postoperatively, the majority of whom had positive stone cultures despite negative preoperative urine cultures [4]. Another study observing flexible ureteroscopy (fURS) and PCNL reported sepsis rates of 0.3% to 7.4% for fURS and 0.9% to 5.9% for PCNL [5]. If this condition is not treated promptly and aggressively, it may even progress to multiorgan failure and death. Consequently, even with a negative urine culture and adequate preoperative antibiotic coverage, postoperative urosepsis may prove catastrophic [6].

As a result, the stone's bacterial flora could serve as a valuable guide for targeted antibiotic therapy in managing postoperative urosepsis [2]. Preoperative urine cultures have been widely used to treat postoperative infections and sepsis, and the use of stone cultures is uncommon in endourologic surgeries [2,4]. There is no clear consensus on the precise prediction of the bacteriology of the stone using the preoperative urine culture. According to the study by Larsen et al., in patients with preoperative bacteriuria, stone cultures revealed bacteria in 77% of the calculi, and they said that sterile urine does not rule out the possibility of bacteriuria [6].

Even when urine cultures are negative, these episodes of sepsis occur. It has been discovered that stones contain bacteria that, when broken up, can result in bacteremia and sepsis. As a result, the usual preoperative urine culture procedure cannot accurately predict the development of postoperative sepsis [7]. Additionally, it seems logical that intraoperative stone cultures could direct us in the prompt treatment of such sepsis episodes. The present study aimed to evaluate the diagnostic and prognostic utility of intraoperative stone culture compared to preoperative urine culture in patients undergoing endourologic procedures for urinary tract stones and to assess its ability to predict postoperative infectious complications and morbidity.

## Materials and methods

A prospective cross-sectional observational study was conducted from June 2023 to December 2024 to evaluate whether stone culture can be used as a predictor of postoperative morbidity. Clearance was obtained from the Institutional Ethical Committee of Bijapur Lingayat District Educational Association, Vijayapura, India (approval number: IEC/998/2022-23).

According to Lang et al. (2022), the incidence of urolithiasis ranges from 1% to 13% globally [4]. For the present study, we assumed a moderate incidence of 6% in the target population. Considering an estimated catchment population size of approximately 20,000 patients attending the tertiary care center during the study period (N = 20,000), the sample size was calculated using the formula:

$n = \frac{Z^{2} \times r (100 - r)}{E^{2}}$

Where Z = 1.96, corresponding to a 95% confidence level, r = 6%, the expected incidence of urolithiasis, E = margin of error (5%).

Substituting these values, the initial required sample size was 87 patients. To compensate for a potential 5% attrition rate, the adjusted minimum sample size was 93 participants. However, to increase the statistical power and ensure robustness of results, we enrolled a total of 125 patients, which not only exceeded the minimum requirement but also improved the precision of estimates and external validity of the study.

All patients aged 18 years or older, presenting during the study period with complaints suggestive of urolithiasis (renal, ureter, or bladder calculi) without prior treatment or instrumentation, were included. Patients on prolonged catheterization or stents, severely immunocompromised, already on broad-spectrum antibiotics before obtaining urine culture, on steroids, and with congenital urologic anatomical anomalies were excluded from the study.

All eligible patients had been explained the study in detail in their own language, and voluntary written informed consent was obtained from all study participants. All patients had undergone complete blood count, urine routine, urine culture and sensitivity (CS), serum creatinine, ultrasound of the kidneys, ureters, and bladder (KUB), and CT KUB. We had analyzed the culture specimens, midstream urine (MSU), and crushed stone culture of eligible patients after endoscopic procedures. Urine cultures were obtained five days prior to scheduled surgery. Patients with negative preoperative urine cultures were administered a single dose of ceftriaxone/ceftazidime (1 g IV) two hours before the procedure, whereas those with positive urine cultures were treated with antibiotics for five days preoperatively according to sensitivity patterns.

Midstream urine was collected with the necessary precautions. Inoculation was done using MacConkey agar and incubated at 37°C for 24 hours. If there was no growth, then the incubated sample was considered sterile. The colony characteristics of the growth from the culture plate were examined after 48 hours and studied for morphology, motility, biochemical tests, and antibiotic sensitivity. Antibiotic sensitivity was done by using the disc diffusion method. The sensitivity of organisms to antibiotics was studied using standard techniques.

Calculi were collected during the procedure in a sterile container and were crushed before being subjected to culture, and the crushed calculus core was cultured in blood agar and MacConkey's agar and incubated at 37°C for 48 hours. The growth from culture plates was examined for the number of colonies. The colony had been studied for morphology, motility, and biochemical reactions. The identification of the bacterial isolate was done by conventional methods. Also, the antibiotic sensitivity of bacterial isolates was determined by using the disc diffusion method.

Patient data was collected as per proforma and entered into Microsoft Excel (Microsoft Corporation, Redmond, WA, USA) and analyzed with IBM SPSS Statistics software, version 26 (IBM Corp., Armonk, NY, USA). Descriptive statistics were presented in numbers and percentages. The association between two non-parametric variables was assessed using the Pearson chi-square test. Continuous variables were presented as mean and SD; a t-test was applied. The diagnostic performance of urine culture was evaluated by calculating sensitivity, specificity, positive predictive value (PPV), negative predictive value (NPV), and overall diagnostic accuracy against intraoperative stone culture findings. A p-value of <0.05 was considered statistically significant.

## Results

The present research included 125 patients with urinary tract calculi. The majority of patients belonged to the age groups of 21-40 years (46, 36.8%) and 41-60 years (44, 35.2%). Most patients had no comorbidity (100 (80.0%)), while diabetes mellitus (17 (13.6%)) and hypertension (six, 4.8%) were the main associated conditions. Left-sided calculi were more common (57, 45.6%) compared to right-sided (51, 40.8%) (Table 1).

**Table 1:** Distribution of patients based on sociodemographic profile, comorbidities, and calculus side Descriptive statistics were presented in numbers and percentages.

Variables	Category	Frequency	Percentage
Age (years)	≤20	10	8.0 %
	21–40	46	36.8 %
	41–60	44	35.2 %
	>60	25	20.0 %
Gender	Male	82	65.6 %
	Female	43	34.4 %
Comorbidities	None	100	80.0 %
	Diabetes mellitus	17	13.6 %
	Hypertension	6	4.8 %
	Other comorbidities	2	1.6 %
Calculus side	Bilateral	4	3.2 %
	Left side	57	45.6 %
	Right side	51	40.8 %
	Vesical	13	10.4 %

Preoperative urine culture was sterile in 98 (78.4%) patients. The most common isolates were *Escherichia coli* (*E. coli*) (16, 12.8%) and *Pseudomonas *(eight, 6.4%). Intraoperative stone culture showed growth in 61 (48.8%) cases, with *Pseudomonas *(21, 16.8%), *E. coli* (19, 15.2%), and *Klebsiella pneumoniae* (nine, 7.2%) as predominant organisms (Table 2).

**Table 2:** Distribution of patients based on urine and intraoperative calculus culture/sensitivity Descriptive statistics were presented in numbers and percentages.

Variables	Category	Frequency	Percentage
Preoperative urine culture/sensitivity	Sterile	98	78.4 %
	Escherichia coli	16	12.8 %
	Pseudomonas	8	6.4 %
	Enterococcus faecalis	1	0.8 %
	Klebsiella	1	0.8 %
	Staphylococcus aureus	1	0.8 %
Intraoperative calculus culture/sensitivity	Sterile	64	51.2 %
	Pseudomonas	21	16.8 %
	Escherichia coli	19	15.2 %
	Klebsiella pneumoniae	9	7.2 %
	Coagulase *Staphylococcus aureus*	4	3.2 %
	Proteus mirabilis	4	3.2 %
	Enterococcus	3	2.4 %
	Candida albicans	1	0.8 %

A comparison between preoperative urine culture and intraoperative stone culture showed no significant relationship (p=0.6091). Sensitivity of urine culture was 19.7%, specificity 76.6%, PPV 44.4%, NPV 50%, and diagnostic accuracy 48.8% (Table 3).

**Table 3:** Cross-tabulation of preoperative urine CS and intraoperative stone CS findings Descriptive statistics were presented in numbers and percentages. The association between two non-parametric variables was assessed using the Pearson chi-square test. The diagnostic performance of urine culture was evaluated by calculating sensitivity, specificity, positive predictive value, negative predictive value, and overall diagnostic accuracy against intraoperative stone culture findings. A p-value of <0.05 was considered statistically significant. CS: culture and sensitivity

Preoperative urine CS	Intraoperative stone CS organism present	Intraoperative stone CS sterile	Total
Organism present	12 (19.7 %)	15 (23.4 %)	27 (21.6 %)
Sterile	49 (80.3 %)	49 (76.6 %)	98 (78.4 %)
Total	61 (100.0 %)	64 (100.0 %)	125 (100.0 %)
Chi-square test = 0.2615; p = 0.6091			

Among patients with sterile preoperative urine cultures, only 49 (50%) had sterile stone cultures, while the remaining grew organisms such as *E. coli* (15, 15.3%), *Pseudomonas* (15, 15.3%), and *Klebsiella* (eight, 8.2%) (Table 4).

**Table 4:** Association between preoperative urine culture and sensitivity findings and intraoperative stone culture sensitivity findings (N=125) Descriptive statistics were presented in numbers and percentages.

Preoperative urine CS	Candida albicans	Coagulase *Staphylococcus aureus*	Escherichia coli	Enterococcus	Klebsiella pneumoniae	Proteus mirabilis	Pseudomonas	Sterile
*Escherichia coli *(n=16)	–	–	3 (18.8 %)	3 (18.8 %)	–	–	–	10 (62.5 %)
*Pseudomonas *(n=8)	–	–	1 (12.5 %)	1 (12.5 %)	–	–	3 (37.5 %)	3 (37.5 %)
*Enterococcus *(n=1)	–	1 (100 %)	–	–	–	–	–	–
*Klebsiella *(n=1)	–	–	–	–	1 (100 %)	–	–	–
*Staphylococcus aureus* (n=1)	–	–	–	–	–	–	–	1 (100 %)
Sterile (n=98)	1 (1.0 %)	3 (3.1 %)	15 (15.3 %)	3 (3.1 %)	8 (8.2 %)	4 (4.1 %)	15 (15.3 %)	49 (50.0 %)
Total	1 (0.8 %)	4 (3.2 % %)	19 (15.2 %)	3 (2.4)	9 (7.2 %)	4 (3.2 %)	21 (16.8 %)	64 (51.2 %)

Patients with microorganisms identified on stone culture showed significantly higher postoperative C-reactive protein (CRP), neutrophil-to-lymphocyte ratio (NLR), total leukocyte count (TLC), and longer hospital stay compared to those with sterile stone cultures (p < 0.05). In contrast, preoperative urine culture results did not show a statistically significant association with these parameters. This comparison highlights the superior predictive value of stone culture in identifying patients at risk for heightened postoperative inflammatory response and prolonged recovery (Table 5).

**Table 5:** Comparison of postoperative parameters with preoperative urine CS and intraoperative stone CS Continuous variables were presented as mean and SD; a t-test was applied. A p-value of <0.05 was considered statistically significant. CS: culture and sensitivity; CRP: C-reactive protein; NLR: neutrophil-to-lymphocyte; TLC: total leukocyte count

Parameters	Preoperative urine CS microorganism (n=27)	Preoperative sterile (n=98)	t value	p value	Stone CS microorganism (n=61)	Stone CS sterile (n=64)	t value	p-value
Postoperative CRP	16.5 ± 13.2	16.4 ± 15.6	0.01	0.990	25.0 ± 14.9	8.3 ± 9.8	7.05	0.001
NLR	3.7 ± 1.7	3.4 ± 1.6	0.89	0.380	4.5 ± 1.2	2.4 ± 1.3	9.05	0.001
Postoperative TLC	12566 ± 3027	12057 ± 3025	0.78	0.440	13753 ± 3055	10656 ± 2076	6.34	0.001
Hospital stay (days)	3.1 ± 1.1	2.9 ± 1.2	0.36	0.717	3.5 ± 1.3	2.5 ± 0.9	4.70	0.001

Patients with positive stone CS had markedly higher morbidity (83.7%) even when urine CS was negative. In our study, “postoperative morbidities” included fever > 38°C, leukocytosis requiring extended antibiotic therapy, urosepsis, and prolonged hospital stay (> 3 days). Conversely, sterile results on both urine and stone CS were associated with very low morbidity (2.0%) (Table 6).

**Table 6:** Postoperative morbidities in relation to urine and stone CS findings (N=125) Descriptive statistics were presented in numbers and percentages. CS: culture and sensitivity

Intraoperative stone CS	Urine CS negative: morbidity present n (%)	Urine CS negative: morbidity absent n (%)	Urine CS positive: morbidity present n (%)	Urine CS positive: morbidity absent n (%)	Total
Negative	1 (2.0 %)	48 (98.0 %)	4 (26.7 %)	11 (73.3 %)	64 (100 %)
Positive	41 (83.7 %)	8 (16.3 %)	9 (75.0 %)	3 (25.0 %)	61 (100 %)

## Discussion

Urinary tract calculi remain one of the commonest urological conditions worldwide, particularly affecting adults between 30 and 60 years of age. In the present study, the majority of patients were aged 21-60 years (72%) and were predominantly male (82, 65.6%). This finding aligns with other Indian studies reporting male predominance and peak incidence between 40 and 60 years [7,8].

Comorbidities in the current study included diabetes mellitus (17, 13.6%) and hypertension (six, 4.8%). Behera et al. reported a higher burden of diabetes (68.8%) and hypertension (50.8%) among elderly patients with UTIs and calculi [9]. Similarly, research from Telangana demonstrated significant associations between renal stone formation and sociodemographic factors; 89% of patients were male, 82% resided in rural areas, and more than 85% belonged to lower or lower-middle socioeconomic classes. Calcium oxalate (pure or mixed) was the predominant stone component (67.9%), and 53.6% of patients had positive urine cultures, with *Klebsiella *(28.6%) and *Proteus *(17.9%) as the most common isolates [10].

In this study, preoperative urine cultures were positive in 27 (21.6%) patients, with *E. coli* (16, 12.8%) and *Pseudomonas* (eight, 6.4%) as the most frequent isolates. This detection rate was lower than that of several other studies. For instance, a study from eastern India reported positive urine cultures in 46% of patients, with *E. coli* as the most common isolate [7]. Similarly, Korean data indicated culture positivity in 74.9% of febrile UTI patients, with *E. coli* (44.1%) and *Pseudomonas *(4.6%) as the leading pathogens [11].

Stone culture has been recognized as more reliable than urine culture in detecting hidden pathogens and guiding targeted antibiotic therapy [2]. In this study, intraoperative stone culture was positive in 61 (48.2%) patients, most commonly isolating *Pseudomonas *(16.8%), *E. coli* (15.2%), and *Klebsiella pneumoniae* (7.2%). European multicenter research has also shown *E. coli* and *Pseudomonas *to be the predominant pathogens in patients with upper urinary tract calculi [12]. A study from southern India similarly highlighted Pseudomonas as the dominant pathogen in stone cultures, even when urine samples were sterile [13]. Another study on 221 patients found a weak correlation between urine and stone cultures, with *E. coli* (30.88%) and *Klebsiella pneumoniae* (19.11%) being the most common isolates. Extended-spectrum beta-lactamase (ESBL) producers were detected in ~37-39% of cases, and methicillin-resistant *Staphylococcus aureus* (MRSA) in ~9%, underscoring the importance of culturing both urine and stones to guide targeted therapy and prevent recurrence [14].

Our findings revealed no significant correlation between preoperative urine and intraoperative stone cultures, consistent with published literature. Gupta et al. observed that urine and stone cultures matched in only 15% of cases, with identical organisms in just 6.5% [15]. Paonessa et al. also noted that 9.7% of patients with sterile urine still had positive stone cultures [16]. Collectively, studies suggest urine culture yields concordant results with stone culture in <30% of cases [17], thus underlining the superiority of stone culture.

The clinical implications are noteworthy. In our study, positive stone cultures were associated with significantly higher postoperative CRP, NLR, TLC, and longer hospital stays, whereas urine culture results showed no such correlation. This confirms that stone cultures are better predictors of postoperative infectious complications. Previous literature corroborates these findings: Margel et al. reported that patients with positive stone cultures had a 3.6-fold increased risk of developing systemic inflammatory response syndrome (SIRS) [17]. Roushani et al. further demonstrated that positive stone cultures, despite sterile urine, were strongly associated with SIRS and prolonged hospitalization, with a tenfold increased risk following PCNL [18].

Several studies also indicate that positive stone culture results necessitate antibiotic modification and predict less favorable outcomes. Korets et al. observed that stone culture positivity was strongly linked with post-PCNL sepsis and elevated CRP and WBC counts even in patients with negative urine cultures [19]. Similarly, Songra et al. reported that 37% of patients with SIRS had positive stone cultures compared to only five patients with positive urine cultures alone, highlighting the prognostic importance of stone culture [3].

In our study, patients with positive stone cultures experienced significantly higher postoperative inflammatory markers (CRP, NLR, TLC) and longer hospital stays compared to those with sterile stones. Importantly, 49 out of 61 patients with positive stone cultures had negative preoperative urine cultures. These patients exhibited substantial postoperative morbidity, including prolonged fever, elevated inflammatory markers, and delayed recovery, whereas patients with both urine and stone cultures negative had minimal morbidity. This demonstrates that stone culture positivity, especially in the context of sterile urine, provides critical prognostic information, allowing clinicians to modify antibiotic therapy postoperatively and better anticipate complications.

The present study has several strengths, including a well-defined cohort, use of both preoperative urine culture and intraoperative stone culture for comparison, and systematic evaluation of postoperative outcomes, which enhance the reliability of findings. However, certain limitations must be acknowledged. The study was conducted at a single center, which may restrict the generalizability of results. Potential confounding factors such as prior antibiotic use, comorbidities, and variations in surgical technique were not fully controlled. Additionally, long-term follow-up for recurrent infections or stone events was not included. Postoperative complications were not graded using a standardized tool such as the Clavien-Dindo classification, which limits the precision and comparability of morbidity assessment. Future multicenter studies with larger cohorts and longer follow-up are warranted to validate and extend these findings.

## Conclusions

Intraoperative stone culture is a superior diagnostic and prognostic tool compared to preoperative urine culture for predicting postoperative infectious complications in patients undergoing endourologic stone procedures. Positive stone cultures, even in patients with sterile urine, are associated with higher inflammatory markers, increased morbidity, and longer hospital stays. Routine incorporation of stone culture in clinical practice can enable early identification of infection, guide targeted antibiotic therapy, and reduce postoperative morbidity, thereby improving overall patient outcomes.
